# Population Pharmacokinetic and Exposure‐Response Analysis of the Cognitive Effects of TAK‐071 in Participants With Parkinson Disease and Cognitive Impairment

**DOI:** 10.1002/cpdd.1579

**Published:** 2025-07-25

**Authors:** Hongxia Jia, Axel Facius, Rachel Jennings, Yaming Hang, Jaya Padmanabhan, Niraj M. Shanbhag, Brian T. Harel, Arthur Simen, Wei Yin

**Affiliations:** ^1^ Quantitative Clinical Pharmacology Takeda Development Center Americas, Inc. Cambridge MA USA; ^2^ thinkQ^2^ AG Baar Switzerland

**Keywords:** cognitive impairment, correlation analysis, exposure–response analysis, Parkinson disease, population pharmacokinetic analysis

## Abstract

TAK‐071 is a novel muscarinic M_1_ positive allosteric modulator under investigation for the treatment of cognitive impairment and falls associated with Parkinson disease (PD). This study evaluated population pharmacokinetics of TAK‐071 following single (1‐160 mg) and multiple (3‐15 mg once daily) oral‐dose TAK‐071 in 112 healthy participants and 53 participants with PD from Phase 1 and Phase 2 studies. A 1‐compartment model with a delayed absorption phase adequately described TAK‐071 pharmacokinetics. Age, body weight, dose, and formulation were significant covariates. Model simulations indicated that age‐adjusted dosing is unnecessary. An exposure‐response relationship on cognitive function (attention, executive function, memory, global) was evaluated. Benefits were observed on attention, executive function, and global cognition, and these plateaued between 5 and 7.5 mg once daily, supporting a dose of 7.5 mg for future clinical studies, as 7.5 mg was well tolerated. As patients with PD can have an increased risk of falls, the relationship between cognitive function and risk of falls, as assessed by stride time variability, was explored. Cognition response for the attention domain score showed consistent and sustained improvement in stride time variability compared with when no response was observed, supporting further investigation of TAK‐071 in PD for the risk of falls.

Parkinson disease (PD) is a progressive neurodegenerative disease characterized by both motor symptoms, most notably tremor, rigidity, and slow movement with a high risk of falls (46%‐68% annual risk),^1^ and nonmotor symptoms, including depression, pain, and cognitive impairment.^1^, ^2^ Affecting more than 1% of people aged 65 years or older, PD is the most common movement disorder and the second most common neurodegenerative disorder after Alzheimer disease.^1^

Cognitive impairment is one of the most common nonmotor symptoms of PD, occurring up to 6 times more often in people with PD than in the healthy population.^2^ Cognitive deficits can arise at any disease stage, and can markedly increase the risk of progression to dementia. People with PD who are diagnosed with mild cognitive impairment (MCI) exhibit subtle cognitive decline, but do not have functional deficits sufficient to meet diagnostic criteria for PD dementia (PDD).^3^ Studies carried out using these criteria have reported frequencies of PD MCI ranging from 14.8% to 42.5% in patients with newly diagnosed PD.^4^, ^5^, ^6^ The cumulative prevalence of PDD is 17% at 5 years, 46% at 10 years, and 83% at 20 years after diagnosis.^7^, ^8^, ^9^

Cognitive impairment in PD is associated with reduced quality of life and a high economic burden.^10^, ^11^ Although there are approved treatments for PDD, there are presently no approved therapies for the treatment of PD MCI. Patients and caregivers indicate that although fall risk can be reduced somewhat through moderate lifestyle changes and use of mobility aids,^12^ cognitive decline is more difficult to control without treatment.^1^

TAK‐071 is a muscarinic M_1_ positive allosteric modulator that selectively activates M_1_ receptors without interfering with the temporal dynamics of acetylcholine (ACh) signaling, which plays a critical role in specific cognitive functions such as attention.^13^ The chemical structure of TAK‐071 has been published previously.^14^ Acetylcholinesterase inhibitors likely cause a tonic increase of ACh, with nonselective direct agonism across muscarinic receptor subtypes and disruption of the temporal dynamics of ACh signaling.^15^ This may obscure the procognitive effects of M_1_ receptor activation. Patients with PD MCI and PDD show a substantial loss of cholinergic fibers.^16^ Consistent with a role of ACh signaling in gait and cognition in PD, there is clinical evidence that cholinergic drugs may reduce fall risk in people with PD, and rivastigmine is approved for PDD.^17^, ^18^, ^19^, ^20^, ^21^ Therefore, a scientific rationale exists for exploring the potential of TAK‐071 to improve cognitive function and reduce fall risk in people with PD.

We conducted preclinical and clinical studies to evaluate the drug metabolism and pharmacokinetics (PK) of TAK‐071. TAK‐071 is mainly metabolized by cytochrome P450 3A4/5 and is not a significant cytochrome P450 3A4/5 inducer or inhibitor. Clinical plasma metabolite scouting indicates no major human metabolites, and urinary excretion of unchanged TAK‐071 is less than 1%. In a Phase 1 clinical study (TAK‐071‐1001) in 143 healthy participants aged 18‐55 years, orally administered TAK‐071 was systemically absorbed and reached maximum concentration (C_max_) 1‐12 hours after administration during multiple dosing. TAK‐071 exhibited a long terminal half‐life (46.3‐60.5 hours) in healthy participants and reached steady state within 14 days of once‐daily dosing. No food effect was observed on TAK‐071 exposure (C_max_ and area under the concentration‐time curve [AUC] from time 0 to infinity).^22^

The objective of this analysis was to characterize the PK of TAK‐071 using a population modeling approach and explore the exposure‐response relationship on cognitive function (attention, executive function, memory, and global) to inform dose selection for future clinical trials in people with PD with cognitive impairment. The relationship between cognitive function and risk of falls, as assessed by stride time variability (STV), was also explored.

## Methods

### Study Design

This population PK analysis included PK data from 2 studies: (1) a Phase 1, escalating single‐ and multiple‐oral‐dose study, with a 3‐way crossover food effect and relative bioavailability study in healthy adults (TAK‐071‐1001; ClinicalTrials.gov, NCT02769065); and (2) a Phase 2, randomized, double‐blind, placebo‐controlled, 2‐period, 2‐treatment (oral 5 or 7.5 mg once daily) crossover study (TAK‐071‐2002; ClinicalTrials.gov, NCT04334317). The Phase 2 study included a sentinel cohort with a single dose in healthy participants aged older than 55 years to evaluate PK in elderly participants before the main study cohort, with multiple doses in participants with PD aged 40‐85 years who had cognitive impairment and an elevated risk of falls. In the main cohort, participants were treated (1:1 randomized to TAK‐071 or placebo) for 6 weeks, followed by a washout period of at least 3 weeks, and then 6 weeks of the crossover treatment. Specific details regarding the clinical study designs of the Phase 1 and Phase 2 studies were described previously.^22^, ^23^ Both studies were approved by an independent institutional review board and conducted in compliance with the ethical principles of the International Conference on Harmonization Tripartite Guideline for Good Clinical Practice. All individuals who participated in the Phase 1 and Phase 2 studies provided fully written informed consent.

### Study Drug Dosing

In the Phase 1 study, single ascending oral doses of TAK‐071 (1, 3, 9, 20, 40, 80, 120, and 160 mg) or matching placebo, and multiple oral doses of TAK‐071 (3, 9, and 15 mg once daily) or matching placebo were administered to healthy participants. In the 15‐mg once‐daily cohort in healthy non‐Japanese participants and in the 3‐, 9‐, and 15‐mg once‐daily cohorts in healthy Japanese participants, an initial single dose was administered, followed by a 7‐day washout, and then multiple doses were administered for 3 weeks. The drug‐in‐capsule formulation was tested in single and multiple ascending‐dose cohorts. In addition, single (10 mg) oral doses of TAK‐071 were administered under a fasted state (drug‐in‐capsule and tablet formulations) or fed state (tablet formulation) to evaluate relative bioavailability and food effect. Food had no impact on TAK‐071 PK.^22^

In the sentinel cohort of the Phase 2 study, a single 7.5‐mg oral dose of TAK‐071 (tablet formulation) or matching placebo was administered to healthy participants, with no restriction on food. Following a preliminary PK analysis of the sentinel cohort, a lower dose of 5‐mg TAK‐071 once daily was selected in the main cohort for participants with PD aged 66‐85 years, whereas participants with PD aged 40‐65 years received 7.5‐mg TAK‐071 once daily. A matching placebo was administered to each age group.

### Sample Collection and Bioanalysis

Blood samples were collected for PK analyses before dosing and at multiple postdose time points up to 168 hours. A detailed description of PK sample collection time points is presented in the Sample Collection Time Points section in the Supplemental Information. Concentrations of TAK‐071 in plasma at each time point were analyzed using validated high‐performance liquid chromatography with tandem mass spectrometry assays. The lower limit of quantification was 1.00 ng/mL. A detailed description of the bioanalytical method is presented in the Bioanalytical Method for Plasma TAK‐071 section in the Supplemental Information.

### Population PK Model Development

The population PK modeling was performed according to a prespecified analysis plan. Nonlinear mixed‐effects models were fitted to PK data using the Nonlinear Mixed Effects Model (NONMEM) program, version 7.5.1 (ICON), compiled with GNU Fortran (Homebrew GCC 12.2.0). Data management, plotting, and tabulation of results were performed in R version 4.2.2 (2022‐10‐31; R Foundation for Statistical Computing). The first‐order conditional estimation with the interaction method was used. A 1‐compartment model was considered for the initial structural model. Because the tail under the semilog plot was straight, a 2‐compartment model was not tested. A delayed absorption consisting of a lag time (*t*_lag_) followed by 3 transit compartments with subsequent fractionated parallel slow and quick absorption and a first‐order elimination process adequately described the TAK‐071 PK. Dose and formulation (tablet and capsule) impacts on the absorption model were incorporated. The parameters estimated were t_lag_, mean transit time (MTT), absorption rate (slow absorption rate [k_a,slow_] and quick absorption rate [k_a,quick_ = k_a,slow_*k_a,q_]), the fraction parameter to split the dose between the slow and quick absorption (frac), relative bioavailability (F_rel_), central compartment clearance (CL), and central volume of distribution (V_c_). Two levels of random effects, namely, between‐subject variability (BSV) and residual unexplained variability, were applied. BSV was implemented using log‐normal error models on MTT, k_a,slow_, CL, and V_c_. The BSV for the *frac* was implemented as a logit‐transformed normal error model. For the residual unexplained variability, a combined additive and proportional error model was used.

A covariate analysis on the TAK‐071 population PK was performed. The purpose of covariate screening was not to identify all statistically significant covariates, but rather to detect major sources of variability. As such, a formal procedure with strict *P* value thresholds was not applied. Instead, covariate candidates were manually selected based on biological plausibility, potential impact on clinical development, and visual inspection of η (ETA) versus covariate plots, supported by *P* values from F‐tests and the slopes of log–log regression lines. Selected candidate relationships were then formally tested in NONMEM and retained if statistically significant (*P* < .05). Covariates investigated were dose, formulation, age, body weight, albumin, alanine transaminase, aspartate transaminase, bilirubin, serum creatinine, estimated creatinine clearance, estimated glomerular filtration rate, race, sex, and individual status (participants with PD vs. healthy participants), and were baseline covariate values. These covariates were initially screened visually for all parameters with random effects (MTT, k_a,slow_, CL, V_c_, and frac). A prescreening for potential covariate effects was performed using F‐tests. For assessing a potential relationship between a continuous variable and a PK parameter, a linear model was fitted to assess the relationship between the corresponding BSV (η) and the logarithm of the covariate candidate, equivalent to a power model. The *P* value (*P* < .05) of the F‐statistic from that model was used to estimate the significance of the reduction of the unexplained variability by this covariate effect. For categorical covariate candidates, an analysis of variance model of the ηs and the covariate categories was considered. Because low to moderate shrinkage was observed in the base model, a manual covariate screening was performed on the basis of plots of individual parameter estimates versus individual characteristics. All equations used in the final population PK model are provided in the NONMEM control stream, which is included in the Supplemental Code. In the covariate model parameterization, body weight was normalized to 80 kg, age to 70 years, and dose to 5 mg. In addition to estimating the primary model parameters, a secondary parameter, terminal half‐life, was derived using the equation ln(2)*Volume/Clearance for comparison purposes.

### Population PK Model Evaluation

The evaluation of the population PK model involved several criteria: biological plausibility, goodness‐of‐fit plots, numerical properties of the model fitting procedure, precision of parameter estimates, shrinkage on the BSV and residual error level, and visual predictive check (VPC).

The goodness‐of‐fit plots included visualizing the predicted plasma concentrations versus observed plasma concentrations; density of residuals, residuals, and population prediction; and density of random effects, scatterplots of random effects versus relevant covariates, and individual fit plots versus time, for each individual separately.

VPCs and/or prediction‐corrected VPCs were generated using 500 simulation trials. The observed median and 5th and 95th percentiles were overlaid with the model‐predicted 90% confidence intervals for each corresponding percentile. This visual comparison was used to assess the agreement between observed and model‐simulated data. If different treatments were summarized in a single VPC, a prediction correction was applied.

### Population PK Model Simulation

The final population PK model was used for simulations. The purpose of the simulations is to assess the effect of age on exposure (AUC during a dosing interval at steady state [AUC_tau,ss_] and C_max_ at steady state [C_max,ss_]) in participants with PD and to support dose selection for future clinical trials. The simulations were based on 100 virtual participants, and 100 simulations per individual were performed, generating 24‐hour profiles at steady state. The simulated 10,000 profiles were used to derive respective AUC_tau,ss_ and C_max,ss_ and the parameters were summarized as geometric means and 80% prediction intervals.

### Exposure‐Response Relationship Analysis

The global cognition score was composed of 3 domains (attention, executive function, and episodic memory) based on 6 performance‐based tests: Sustained Attention Test, Symbol Digit Modalities Test, Cogstate Groton Maze Learning Test, Cogstate One Back Test, Cogstate International Shopping List Test, and Cogstate One Card Learning Test.^23^ The International Shopping List Test generated 2 scores: an immediate recall score and a delayed recall score. The global cognition score was computed as the mean of the standardized *z*‐scores of these tests, but required that at least 5 to 7 scores were available.^23^ Individuals’ global cognition scores and each domain score change from baseline at Week 6 were plotted versus individual plasma concentrations at the end of the dosing interval at steady state (C_trough,ss_), C_max,ss_, and AUC_tau,ss_ for participants with PD from TAK‐071‐2002. These individual PK exposures were fixed to individual empirical estimates from the final population PK model. For participants receiving placebo, the PK exposures were set to 0. Quartile plots of change from baseline in global cognition score and the 3 domains (executive function domain score, attention domain score, and episodic memory domain score) following 6 weeks of treatment with TAK‐071 or placebo were plotted versus C_trough,ss_, C_max,ss_, and AUC_tau,ss_. Direct response models, including linear, saturation, and reverse “U”‐shape models, were explored (Supplemental Equation 1).

### Analysis of Correlation of Efficacy End Points

To confirm the relationship between cognition and risk of falls, and their shared cholinergic underpinning, an additional analysis was undertaken using the Phase 2 data. Cognition was measured using the 6 previously described tests; these tests were used to calculate 3 domain scores (attention, executive function, and episodic memory) and a global score. STV is a gait metric reflecting the average variability of the left and right foot stride time and is associated with risk of falls. Participants were asked to wear 6 motion sensors located on the feet, wrists, sternum, and lumbar back during a natural‐pace 2‐minute walk on a 10‐m walkway.^23^ STV was measured using these sensors and was assessed both with and without a serial 3 subtraction test to incorporate cognitive loading, known to increase STV.^24^, ^25^, ^26^ STV was recorded at baseline and after 6 weeks of treatment with either TAK‐071 or placebo.

Irrespective of treatment, changes from baseline on the cognitive domain and global scores were used to define cognitive response and no response groups using a threshold (τ), that is, change from baseline for each of the domain and global scores greater than τ (where τ is greater than 0) indicated a response, and change from baseline for each of the domain and global scores of τ or less indicated no response. τ was varied, with larger values reflecting a greater cognitive improvement with respect to the score being used. At each value of τ, the mean (±standard error) was calculated for both groups, and statistical significance was assessed using the 1‐sided Wilcoxon rank‐sum (also known as Mann‐Whitney U) test to determine if the mean change from baseline in STV for the response group was less than that for no response, indicating that an improvement in a cognitive domain or global cognition (ie, a response) was accompanied by a reduction in STV. Vargha and Delaney's A, an effect size representing the probability that an observed change from baseline in STV for the cognitive response group will be less than that for no response,^27^ was also computed.

## Results

### Data Set

The population PK data set consisted of 104 healthy participants from TAK‐071‐1001 (treated with a single dose between 1 and 160 mg and multiple doses between 3 and 15 mg once daily) as well as participants from TAK‐071‐2002 (8 healthy participants received a single dose of 7.5 mg from the sentinel cohort of TAK‐071‐2002; 37 participants with PD received 5‐mg once‐daily doses and 16 participants with PD received 7.5‐mg once‐daily doses from the main cohort). The population of study TAK‐071‐1001 was mostly men (96%) and aged between 18 and 55 years; 16% were Japanese participants. Study TAK‐071‐2002 was conducted in a more elderly population, aged between 56 and 83 years; approximately 25% were women, and there were no Japanese participants. Additional demographic characteristics of participants in the population PK analysis are provided in Table S1. The demographic characteristics for the exposure‐response analysis have been described previously.^23^

### Population PK Analysis

**Figure 1:**
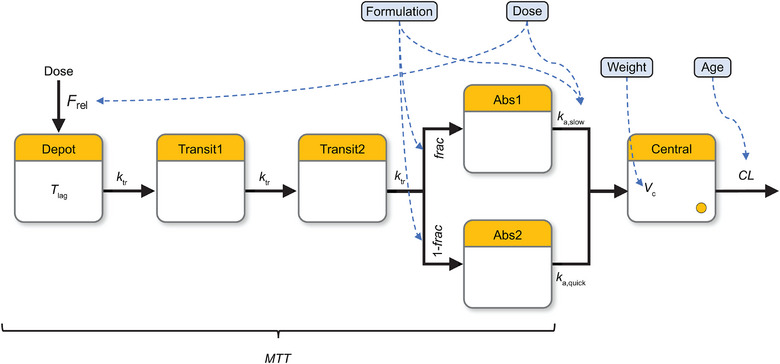
Final population pharmacokinetic model structure with covariate effects. The blue dashed lines represent the covariates identified in the final model: dose was identified as a covariate on relative bioavailability and slow absorption rate, formulation on fraction (of slow and fast absorption) and slow absorption rate, age on clearance, and body weight on volume of distribution. Abs1, slow absorption compartment; Abs2, quick absorption compartment; CL, clearance; frac, fraction absorbed slowly; F_rel_, relative bioavailability; k*_a,_*_slow_, slow absorption rate; k*_a_*_,quick_, quick absorption rate; k*_tr_*, transit rate; MTT, mean transit time; T_lag_, lag time; V_c_, volume of distribution.

The structure of the final population PK model is presented in Figure 1. The final base model was described in the Methods section. Significant covariates identified to impact TAK‐071 PK include dose on relative bioavailability and slow absorption rate, formulation on fraction (of slow and fast absorption), and slow absorption rate, age on clearance, and body weight on volume of distribution.

Relative bioavailability decreases as the dose increases. The absorption rate slows as the dose increases. The tablet formulation has a lower slow absorption fraction compared with the capsule formulation. The tablet formulation also has a higher absorption rate than the capsule formulation. Clearance decreases with age, while the volume of distribution increases with body weight.

Albumin, alanine transaminase, aspartate transaminase, bilirubin, serum creatinine, estimated creatinine clearance, estimated glomerular filtration rate, race, sex, and individual status (patients vs healthy participants) were not identified as significant covariates on TAK‐071 PK.

Parameter estimates for the final population PK model are provided in Table 1. All parameters were estimated with high precision. Low to moderate η‐shrinkage of the random‐effect parameters as well as the ε‐shrinkage of the residuals were observed. The terminal half‐life estimated by noncompartmental analysis ranged from 46.3 to 60.5 hours in the Phase 1 cohorts and was 70.7 hours in the Phase 2 sentinel cohort. The model‐estimated terminal half‐life was 58 hours, falling within this observed range. This consistency confirms that the model adequately captured the data, supporting the chosen compartmental structure. The final model showed a delay between the dosing time and the time of the first quantifiable PK concentrations in plasma. This delay was characterized by an MTT (including a fixed t_lag_ of 0.2 hours), estimated at 1.54 hours, with a percent coefficient of variation (CV%) of 69.8% assessed for interindividual variability (IIV; derived from BSV of 63.0% based on Supplemental Equation 2). After this delay, the drug levels rose quickly in plasma. The complex absorption could be well characterized by a mixture of 2 absorption routes. The slower absorption route (approximately 80.4% of the dose was absorbed by this route) had a k_a,slow_ of 0.883 L/h and the faster absorption route had a k_a,quick_ of 62.7 L/h. The V_c_ was estimated at 47.5 L with a CV% of 23.6% for IIV, and the CL was estimated at 0.566 L/h with a CV% of 38.6% for IIV. The sparse PK data collected in patients with PD did not allow for reliable estimation of individual absorption rates and fraction parameters, which is why no random effects for k_a_ and frac were estimated for this subset. In addition, the model could quantify an inflated residual variability in this subset of approximately 21%. According to a visual inspection of the scatterplots between transformed BSV (η, the discrepancy of an individual parameter from the typical population value) and covariates, and guided by the F‐test results (low *P* values) and the slopes (sufficient magnitude) of the linear regression models described in the Methods section, the following covariates were identified and selected for subsequent formal NONMEM‐based testing: age on CL, body weight on V_c_, and estimated creatinine clearance on CL. Aspartate transaminase was not tested on MTT due to the unlikely biological plausibility and minor importance of the MTT parameter.

**Table 1:** Final Population Pharmacokinetic Model Parameter Estimates

Parameter	Role	Estimate	RSE	95% CI
MTT	TV (hour)	1.54	2.3	1.47 to 1.61
Fraction absorbed slowly	TV (%)	80.4	0.5	79.7 to 81.2
	Tablet‐effect (ratio)	0.419	5.8	0.372 to 0.467
Absorption rate	TV^b^ (1/h)	0.883	1.9	0.851 to 0.916
	Dose‐effect (power)	−0.885	5.7	−0.984 to −0.787
	Tablet‐effect (ratio)	27.0	7.6	23.0 to 31.0
Absorption ratio quick/slow	TV (ratio)	71.0	1.9	68.3 to 73.7
Clearance	TV^c^ (L/h)	0.566	2.2	0.542 to 0.591
	Age‐effect (power)	−0.171	37.9	−0.298 to −0.0439
Volume	TV^d^ (L)	47.5	2.7	45.0 to 50.0
	Body weight‐effect (power)	0.913	13.8	0.666 to 1.16
Relative bioavailability	Dose‐effect (power)	−0.0965	13.8	−0.123 to −0.0705
MTT	BSV^a^	0.630	2.8	0.596 to 0.665
Fraction absorbed slowly	BSV^a^	1.13	3.8	1.05 to 1.21
Absorption rate	BSV^a^	0.788	4.0	0.727 to 0.849
Clearance	BSV^a^	0.373	3.2	0.349 to 0.396
Volume	BSV^a^	0.233	2.6	0.221 to 0.245
RUV	Prop. (%)	11.5	0.6	11.3 to 11.6
	Add. (ng/mL)	5.29	1.5	5.14 to 5.44
	Sparse (ratio)	1.21	3.4	1.13 to 1.29

Add., additive residual error; BSV, between‐subject variability; CI, confidence interval; MTT, mean transit time; Prop., proportional residual error; RSE, relative standard error; RUV, residual unexplained variability; TV, typical value.

a Results for random‐effect parameters are shown as standard deviations/correlations as reported by the Nonlinear Mixed Effects Model program.

b Typical value for a 5 mg dose with formulation DIC.

c Typical value for a person aged 70 years.

d Typical value for a person with 80 kg body weight.

**Figure 2:**
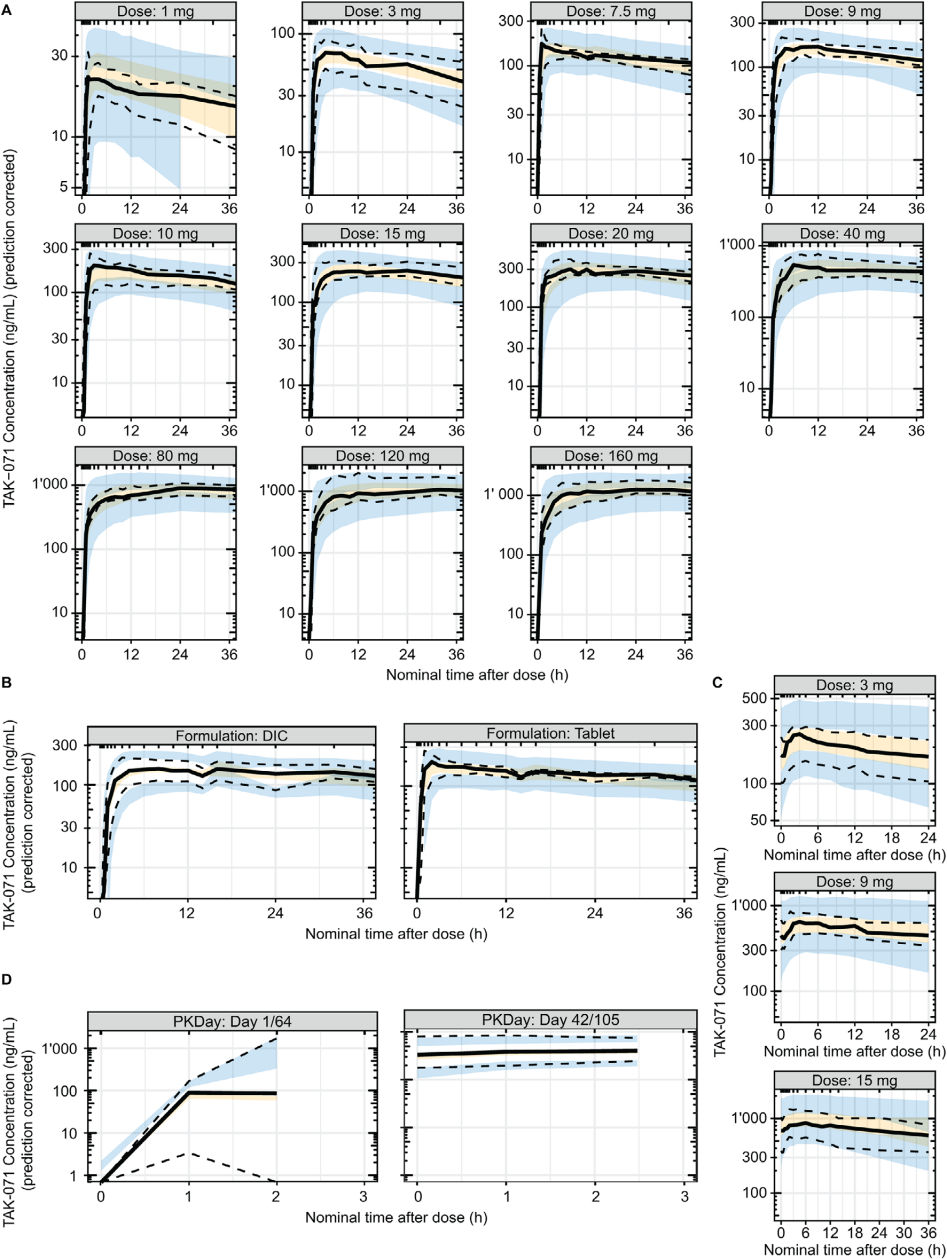
Prediction‐corrected VPC plots for the population PK model of plasma TAK‐071. The black solid line represents the median observed concentrations. The black dashed lines represent the 5th and 95th percentiles of the observed concentrations. The orange shaded band represents the 90% confidence intervals of the medians of the simulated concentrations across the 500 simulation trials. The blue shaded bands represent the 90% prediction intervals of the simulated concentrations across the 500 simulation trials. (A) Prediction‐corrected VPC by dose level, all single‐dose profiles in TAK‐071‐1001 and TAK‐071‐2002, densely sampled participants only. (B) Prediction‐corrected VPC by formulation, all single‐dose profiles in TAK‐071‐1001 and TAK‐071‐2002, densely sampled participants only. (C) VPC by dose of study TAK‐071‐1001, repeated dose profiles, densely sampled participants only. (D) Prediction‐corrected VPC by day, sparsely sampled PD participants in TAK‐071‐2002. DIC, drug in capsule; PK, pharmacokinetic; VPC, visual predictive check.

Goodness‐of‐fit plots for the final population PK model indicated a good fit to the observed data (Figure S1). The final population PK model adequately described the plasma concentration of TAK‐071 in healthy participants and participants with PD. The prediction‐corrected VPC plots show that the median (solid line) as well as the 5th and 95th percentiles (dashed lines) of the observed concentrations were, in most cases, within the model‐predicted 90% confidence intervals (shaded bands, orange for the median, blue for the 5th and 95th percentiles), therefore supporting the suitability of the final model (Figure 2). A larger IIV in the absorption phase on Day 1 in participants with PD, indicated by the prediction‐corrected VPC, may be due to the limitation of sparse PK sampling. Overall, the VPC looks good, indicating that the population PK model is adequate.

For the sentinel cohort of the Phase 2 study, the plasma PK parameters of TAK‐071 following a single oral administration of 7.5 mg TAK‐071 in healthy participants aged 56‐74 years were calculated using a noncompartmental analysis and summarized in Table 2. To match the sentinel cohort PK exposure, a lower dose was administered to elderly participants in the subsequent main cohort: 5 mg once daily in participants with PD aged 66‐85 years, inclusive. Participants with PD aged 40‐65 years, inclusive, received the same dose as the sentinel cohort. The plasma PK parameters of the main cohort were estimated using the final population PK model and summarized in Table S2.

**Table 2:** Summary of Plasma Pharmacokinetic Parameter Estimates of TAK‐071 Following Single Oral Administration of 7.5‐mg TAK‐071 to Healthy Individuals: Sentinel Cohort

	t_max_ (hour)	C_max_ (ng/mL)	AUC_∞_ (ng•h/mL)	t_1/2z_ (hour)
Mean^a^	1.50	195	15,100	70.7
SD^b^	1.00‐8.00	59.0	4080	23.1
CV	–	30.3	26.9	32.6

AUC_∞_, area under the concentration‐time curve to infinity; C_max_, maximum observed plasma concentration; CV, coefficient of variation; SD, standard deviation; t_1/2z_, terminal disposition phase half‐life; t_max_, time to reach C_max_.

a Median is presented for t_max_.

b Minimum‐maximum is presented for t_max_.

Based on the final population PK model, while age is a statistically significant covariate for central compartment clearance, the age‐effect estimate of −0.171 in the power model suggests that the effect of age on central compartment clearance is minimal. Additionally, the difference in simulated TAK‐071 PK exposure (C_max,ss_ and AUC_tau,ss_) for people with PD aged 40 and 85 years, compared with the reference age of 70 years was less than 10%. This suggests that age has a minimal effect on exposure; therefore, no dose adjustment by age should be necessary. Because nonclinical data suggested that the mean PK exposure should be kept below a C_max,ss_ of 600 ng/mL and an AUC_tau,ss_ of 14,000 ng•h/mL, the highest dose that can be administered to participants aged 40 and 85 years is 7 mg once daily, which provides the model‐predicted geometric mean (80% prediction interval) C_max,ss_ of 562 (374‐863) ng/mL and AUC_tau,ss_ of 11,900 (7410‐19,200) ng•h/mL. The difference between the simulated PK exposure of TAK‐071 in people with PD with a body weight of 120 and 50 kg and the reference body weight of 80 kg was within 10% (Figure S2), confirming adequacy of a flat dose (ie, no dose adjustment should be needed by body weight).

### Exposure‐Response Relationship Analysis

**Figure 3:**
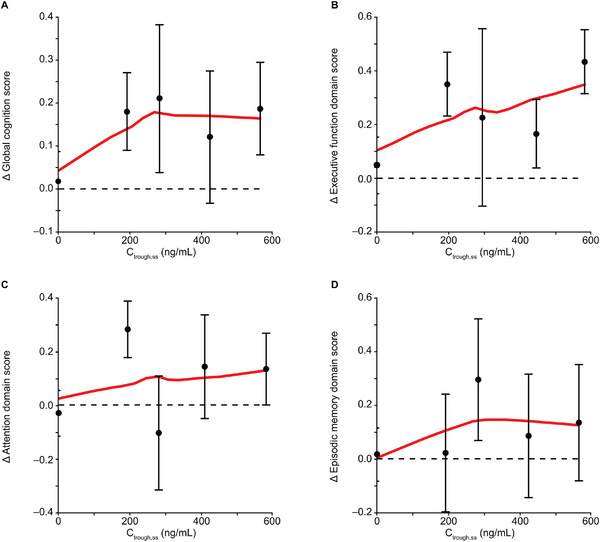
Quartile plot of global cognition score and domain scores versus C_trough,ss_. (A) Change from baseline in global cognition score versus C_trough,ss_. (B) Change from baseline in executive function domain score versus C_trough,ss_. (C) Change from baseline in attention domain score versus C_trough,ss_. (D) Change from baseline in episodic memory domain score versus C_trough,ss_. Mean (± standard error) scores change from baseline following 6 weeks of placebo and each quartile of the C_trough,ss_ following 6 weeks of TAK‐071 treatment were presented. The red lines represent the locally estimated scatterplot smoothing regression lines of the mean values. Δ, change from baseline; C_trough,ss_, plasma concentration at the end of the dosing interval at steady state.

Participant compliance was high. Mean (standard deviation) study medication compliance (% of days) was 98.9% (2.37%) for participants treated with placebo and 98.9% (2.94%) for participants treated with TAK‐071. In the exposure‐response analysis, positive trends on global cognition score and executive function domain score at Week 6 versus steady‐state PK exposure parameters were identified. The global cognition score and executive function domain score change from baseline at Week 6 versus the C_trough,ss_ quartile plot showed that the score change from baseline was close to 0 for placebo and reached a plateau between 200 and 800 ng/mL of C_trough,ss_ (Figure 3). The plateaued range of C_trough,ss_ is comparable to the range of C_trough,ss_ following 5 and 7.5 mg once daily, being 167‐725 ng/mL and 211‐743 ng/mL, respectively, indicating that the clinical benefit plateaued between 5 and 7.5 mg once daily. In addition, this C_trough,ss_ range is higher than the in vitro half‐maximal effective concentration of the Ca^2+^ mobilization assay (100 ng/mL), which represents the human M_1_ receptor activity, and is comparable with the range of the efficacious concentrations determined from rat and mouse cognition models (72‐438 ng/mL), providing an understanding of the observed clinical efficacy at these doses. Minor trends were observed for the attention domain score and episodic memory domain score. The trends for different PK parameters (C_max,ss_ and AUC_tau,ss_) were similar to those observed for C_trough,ss_ (Figure S3). Although there is no differentiation among the 4 quartiles across the concentration range, statistical analysis comparing the treatment group (combining all 4 quartiles) with the placebo group showed statistically significant improvements in global cognition score (*P* < .05) and executive function (*P* < .01) after TAK‐071 treatment compared with placebo.^23^

Direct response models, including linear, saturation, and reverse “U”‐shape models were tested for the relationship between change from baseline in global cognition score at Week 6 versus C_trough,ss_ but resulted in a large relative standard error of parameter estimates, likely due to a lack of data points at C_trough,ss_ lower than approximately 150 ng/mL.

### Analysis of Correlation Between Efficacy End Points

**Figure 4:**
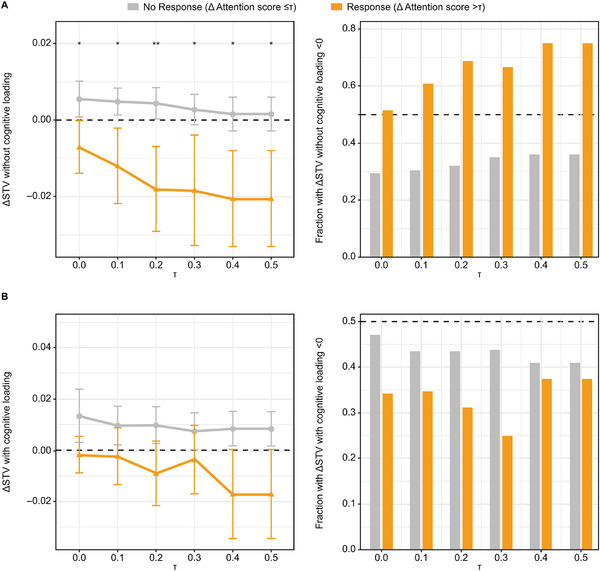
Differences in change from baseline in STV (A) without cognitive loading and (B) with cognitive loading when change from baseline in attention score is used to define cognitive response and no cognitive response groups across varying τ. The panels on the left display the mean ± standard error, with * and ** indicating statistical significance for apparent *P* values of <.05 and <.01, respectively. Testing examined if the mean change from baseline in STV in the no cognitive response group was less than that for no response, indicating an improvement in STV for those who also had an improvement in cognition. The panels on the right provide the fraction of the cognitive response and no response groups exhibiting an improvement in STV (ie, change from baseline in STV <0). Δ, change from baseline; STV, stride time variability.

An analysis was conducted using Phase 2 data to evaluate the relationship between cognition and STV with or without cognitive loading. When the attention domain score was used to define data points exhibiting a cognitive response (change from baseline in attention greater than τ) and no response (change from baseline in attention of τ or less) across varying thresholds (τ), the differences in mean values for change from baseline in STV without cognitive loading showed clear delineation, with mean change‐from‐baseline (Δ) STV for the cognitive response group being both less than 0 and less than the mean ΔSTV for the no cognitive response group across increasing τ. Moreover, the difference between the means with that of the response group being less than that for no response were found to be statistically significant (apparent *P* values of less than .05 or .01; Figure 4A) for all values of τ. This suggests that a reduction in STV (ΔSTV less than 0) may correlate with an improvement in attention score. Majority of data points in the response group (>50%) exhibited an improvement in change from baseline in STV without cognitive loading (ie, change from baseline in STV less than 0) compared with fewer than 40% for no response (Figure 4A), suggesting distributional differences between the 2 groups. These findings are supported by the small to moderate effect sizes detected (Table S3).

Similar results were found for the mean change from baseline in STV with cognitive loading; however, the difference between cognitive response and no response groups was not statistically significant for all τ (Figure 4B). For the attention score response group, change from baseline in STV with cognitive loading was no longer strongly skewed toward showing an improvement (Figure 4B). On the other hand, small effect sizes (ie, a greater likelihood of change from baseline in STV, with cognitive loading for the response group being less than that for no response) were identified for τ of 0.4 and 0.6.

An improvement in cognition did not coincide with a reduction in STV for the analyses of change from baseline in STV without cognitive loading when a response/no response was defined using the executive function domain score, or for change from baseline in STV with cognitive loading with response/no response defined using the episodic memory domain score. For the remaining combinations, the relationship between the 2 groups was found to be inconsistent or similar to that identified for the attention domain with respect to change from baseline in STV, with 1 exception: When cognition response was defined using the executive function domain score, the mean change from baseline in STV with cognitive loading was found to be less than that of the no response group at the largest value of τ that was statistically significant (apparent *P* value of less than .05) and with the distribution of change from baseline in STV skewed negative (ie, showing an improvement). Analogous figures for executive function domain, episodic memory domain, and global cognition scores are provided in Figure S4, with sample sizes, effect sizes, and apparent *P* values reported in Table S3.

## Discussion

This study is the first to characterize the PK of TAK‐071 in healthy participants and participants with PD using a nonlinear mixed‐effects PK model. TAK‐071 was shown to exhibit the characteristics of a Biopharmaceutics Classification System Class II compound (insoluble in water and high permeability), resulting in complex absorption profiles following oral doses. The absorption profiles were adequately described by a 1‐compartment linear model with 2 parallel first‐order absorption routes, characterized by a slow and fast absorption rate. Both absorptions were delayed using a chain of transit compartments. This complex absorption model was required to adequately characterize the 2 formulations (tablets and capsules), where the capsules showed a prolonged absorption phase with dose‐dependent changes.

The plasma protein binding of TAK‐071 was high (98.9% or greater, ie, unbound fraction of 1.1% or less), and urinary excretion of TAK‐071 was minimal. In the Phase 1 study, after a single oral administration of TAK‐071 (1 to 160 mg), minimal urinary excretion was observed, with a mean total recovery in urine of less than 0.3% of the dose. Following multiple oral administrations of TAK‐071 (3‐15 mg once daily for 21 days) in both non‐Japanese and Japanese individuals, minimal urinary excretion was again observed, with a mean total recovery of less than 0.8% of the dose for both groups. In the population PK covariate analysis, both estimated creatinine clearance and eGFR were tested, but neither was a significant covariate for clearance. This is consistent with clinical observations.

Based on observed Phase 1 results, under the fasted state following a single 10 mg dose, TAK‐071 systemic exposures (C_max_ and AUC from time 0 to infinity) for the tablet and capsule formulation were not significantly different.^22^ However, the median time of first occurrence of C_max_ was delayed for the capsule formulation versus tablet formulation (4.98 and 2.00 hours, respectively). Based on the population PK covariate analysis, formulation is a significant covariate for the fraction of slow and fast absorption, as well as the slow absorption rate. This is consistent with clinical observations.

Although in the Phase 2 study elderly participants aged 66‐85 years received a lower dose (5 mg once daily) than participants aged 40‐65 years (7.5 mg once daily), our analyses support administration of the same TAK‐071 dose irrespective of age (up to 7 mg once daily for the whole population aged up to 85 years) because the difference in simulated PK exposure was less than 10% for participants aged 40 and 85 years versus 70 years.

An exposure‐response relationship analysis found trends toward improvement in global cognition and executive function scores with greater TAK‐071 exposure, with response plateauing between 5 and 7.5 mg once‐daily TAK‐071. Together with a favorable safety profile up to 7.5 mg once daily in participants with PD, this further supports 7 mg once daily as the adequate dose to evaluate efficacy in future clinical trials in participants up to age 85 years.

C_trough,ss_ was selected as the primary exposure metric for the exposure–response analysis based on 2 key considerations. First, C_trough,ss_ is directly measurable and less dependent on the timing of sample collection compared to AUC, which can introduce potential bias. Second, M1 receptor activation occurs rapidly, within seconds,^28^ although the exact timing of the cognitive effect remains unclear. Using C_trough,ss_ allows us to explore the potential minimum concentration needed to sustain cognitive effects. For a more comprehensive assessment, additional exposure metrics, including C_max,ss_ and AUC_tau,ss_, were also evaluated in the exposure‐response analysis (Figure S3). Although the exposure‐response models that we explored were based on a small dose range of 5‐7.5 mg once daily, such modeling approaches can support dose selection for future clinical trials.

When the attention domain score was used to define cognitive response and no response, irrespective of treatment, an improvement in attention (response) coincided with an improvement in STV. This relationship was found to be statistically significant for STV without cognitive loading. It should be noted that the Wilcoxon rank‐sum test assumes independence between the response and no response in cognition groups, which may not hold true for all threshold values, especially considering the crossover design of the Phase 2 study. More data would be required to investigate this effect further. When the executive function domain, episodic memory domain, and global cognition scores were examined, similar findings, though to a lesser degree, were identified for change from baseline in STV either with or without cognitive loading, but not both. This could be attributable to the limited number of data points, which diminished dramatically for larger thresholds that indicate greater improvement in the executive function domain, episodic memory domain, and global cognition scores. The consistent correlation between improved attention scores and reduced STV suggests a shared involvement of the cholinergic pathway in these outcomes, supporting the rationale for exploring the potential of TAK‐071 to improve cognition (in particular, attention) and decrease the risk of falls in patients with PD.^29^, ^30^ This also underscores the appropriateness of using the Cogstate cognitive assessments to assess cognition and STV as a metric for risk of falls.

This study has several limitations. First, a bootstrap analysis was not performed due to the high computational demands, although the population PK model demonstrated a good fit to the observed data. Second, the number of data points available for participants with PD was limited within each quartile of the exposure‐response analysis. Third, data at lower exposure levels were unavailable because lower doses were not tested. In future studies, evaluating lower doses (eg, 0.5 mg once daily or 3.5 mg once daily) may help better define the shape of the exposure‐response curve, particularly given that the response appeared to plateau at 5 mg once daily.

## Conclusion

We found that our final population PK model adequately described TAK‐071 PK in healthy participants and participants with PD. Based on our model, age was found to have a minimal effect on exposure, suggesting that TAK‐071 does not require dose adjustment for age. Exposure‐response relationship analyses of TAK‐071 identified positive trends in cognitive function and a correlation between improved attention and reduced risk of falls. These analyses will help inform TAK‐071 dose selection for future clinical trials.

## Conflicts of Interest

Hongxia Jia, Rachel Jennings, Yaming Hang, Brian T. Harel, Arthur Simen, and Wei Yin are employees of Takeda Pharmaceutical Company Ltd and own stock or stock options. Jaya Padmanabhan and Niraj M. Shanbhag were employees of Takeda Pharmaceutical Company Ltd at the time of the study and owned stock or stock options. Axel Facius is a former employee of Takeda and received payment for acting as a consultant for Takeda at the time of data analysis.

## Funding

This study was sponsored by Takeda Development Center Americas, Inc. (Cambridge, MA, USA), and the Michael J. Fox Foundation, grant ID 17922.

## Supporting information



Supporting Information

## Data Availability

The data sets, including the redacted study protocol, redacted statistical analysis plan, and individual participants' data supporting the results reported in this article, will be made available, within 3 months from initial request, to researchers who provide a methodologically sound proposal. The data will be provided after deidentification, in compliance with applicable privacy laws, data protection, and requirements for consent and anonymization.
