# Supplementary material for: Population Pharmacokinetic and Exposure‐Response Analysis of the Cognitive Effects of TAK‐071 in Participants With Parkinson Disease and Cognitive Impairment
Source: Clin Pharmacol Drug Dev. 2025 Jul 25;14(11):856–68. doi: 10.1002/cpdd.1579 (PMC12583984; doi:10.1002/cpdd.1579)
Supplement: Supplementary file 1 — Supporting Information [file CPDD-14-856-s001.pdf]

Supplemental Information

Population Pharmacokinetics and Exposure–Response Analysis of the Cognitive Effects of TAK-071 in Participants With Parkinson Disease and Cognitive Impairment

Hongxia Jia[1], Axel Facius[2], Rachel Jennings[1], Yaming Hang[1], Jaya Padmanabhan[1], Niraj M. Shanbhag[1], Brian T. Harel[1], Arthur Simen[1], and Wei Yin[1]

[1]Takeda Development Center Americas, Inc, Cambridge, MA, USA
[2]thinkQ[2] AG, Oberneuhofstrasse 5, 6340 Baar, Switzerland

Corresponding Author:

Wei Yin, PhD, Takeda Development Center Americas, Inc., 35 Landsdowne St, Cambridge, MA 02139, USA (phone: +1 617-679-7000; email: wei.yin@takeda.com; orcid.org/0000-0002-4834-5783)

Table of Contents

Sample Collection Time Points 2
Bioanalytical Method for Plasma TAK-071 3
Supplemental Figures 4
Supplemental Tables 9
Supplemental Equations 14
Supplemental Code. NONMEM Control Stream of the Final Model 15

Sample Collection Time Points

In the Phase 1 study, the following serial blood samples for determination of plasma TAK-071 concentration were collected in healthy volunteers: (1) before TAK-071 administration (pre dose) on Day 1 and at 0.25, 0.5, 1, 1.5, 2, 3, 4, 6, 8, 10, 12, 14, 24, 48, 72, 96, and 168 hours post dose in the single ascending dose cohorts receiving 1–80 mg; (2) pre dose and at 0.5, 1, 2, 4, 6, 8, 10, 12, 16, 32, 40, 48, 56, 64, 72, 96, and 168 hours post dose in the cohort receiving a single dose of 120 and 160 mg; (3) pre dose and at 0.25, 0.5, 1, 1.5, 2, 3, 4, 6, 8, 10, 12, 14, and 24 hours post dose on Day 1; pre dose on Day 8, 14, 19 and 20; and pre dose and at 0.25, 0.5, 1, 1.5, 2, 3, 4, 6, 8, 10, 12, 14, and 24 hours post dose on Day 21 in the 3- and 9-mg once-daily (QD) cohorts in healthy non-Japanese volunteers; (4) pre dose and at 0.25, 0.5, 1, 1.5, 2, 3, 4, 6, 8, 10, 12, 14, 24, 48, 72, and 96 hours post dose on Day 1; pre dose and at 0.25, 0.5, 1, 1.5, 2, 3, 4, 6, 8, 10, 12, 14, and 24 hours post dose on Day 8; pre dose on Day 15, 20, 26, and 27; pre dose and at 0.25, 0.5, 1, 1.5, 2, 3, 4, 6, 8, 10, 12, 14, 24 and 36 hours post dose on Day 28 in the 15-mg QD cohort in healthy non-Japanese volunteers; (5) same time points as in (4) except the 36 hours post dose on Day 28 was not collected in the 3-, 9-, and 15-mg QD cohorts in healthy Japanese volunteers; (6) pre dose and at 0. 5, 1, 2, 3, 4, 6, 8, 10, 12, 16, 24, 32, 40, 48, 72, 96, and 168 hours post dose on Day 1 of each period of the food effect and relative bioavailability cohort.

In the Phase 2 study, the following serial blood samples for determination of plasma TAK-071 concentration were collected: (1) pre dose and at 0.5, 1, 2, 3, 4, 6, 8, 10, 12, 14, 24, 48, 72, 96, and 168 hours post dose in the sentinel cohort in healthy volunteers; (2) pre dose and at 1 and 2 hours post dose on Day 1 and pre dose and at 1, 2, and 3 hours post dose on Day 42 in each period in the main cohort in people with Parkinson disease with cognitive impairment and an elevated risk of falls.

Bioanalytical Method for Plasma TAK-071

The internal standard was deuterated TAK-071 (TAK-071-d_{11}). Analytes were isolated through protein precipitation extraction. The HPLC analytical column was Waters XBridge C18, 3.5 µ, 2.1 x 100 mm. Mobile phase A was 10 mM ammonium formate/formic acid (1000:2, v/v). Mobile phase B was acetonitrile. MS instrument was SCIEX API 4000. The settings were:

Resolution Q1: Unit

Resolution Q3: Unit

Acquisition time: 5 min

Curtain gas flow (CUR): 30

Gas supply 1 (GS1): 60

Gas supply 2 (GS2): 70

Ion spray voltage (IS): 4000 V

Source temperature (TEM): 600 °C

Collision gas flow (CAD): Medium

Instrument settings for each analyte are shown in the table below:

Analyte	~ Rt (min)	Dwell time (ms)	Q1 m/z	Q3 m/z	DP	EP	CE	CXP
TAK-071	1.63	300	422.3	334.2	65	10	30	15
TAK-071-d_{11}	1.60	100	433.2	345.2	70	10	32	15

CE, collision energy; CXP, collision exit potential; DP, declustering potential; EP, entrance potential; m/z, mass/charge; Q1, first quadrupole; Q3, third quadrupole; Rt, retention time.

This method is applicable to the quantitation of TAK-071 within a nominal range of 1.00 ng/mL to 1500 ng/mL. The within-day precision was 2.77% to 7.70% and accuracy was −8.86% to 3.93%. The between-day precision was 4.36% to 6.77% and accuracy was −5.59% to −0.526%.

Supplemental Figures

A

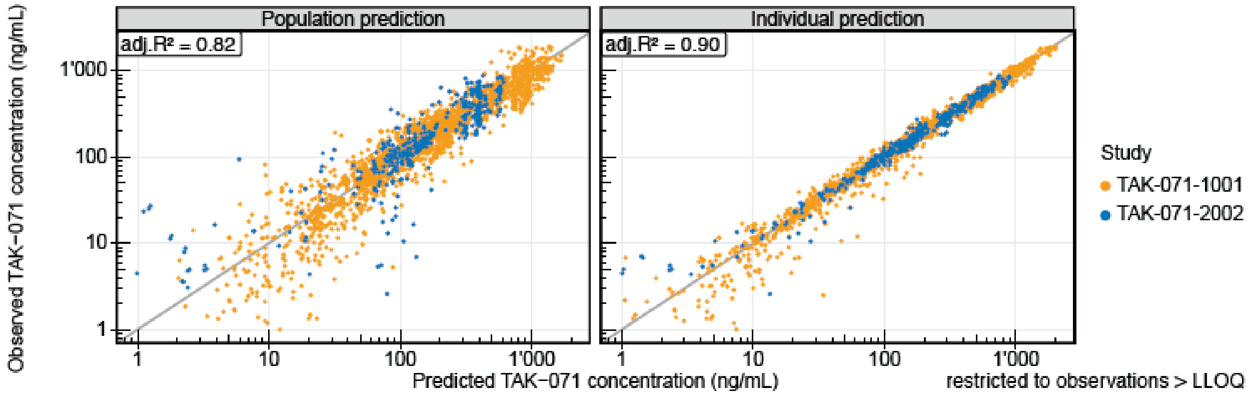

B

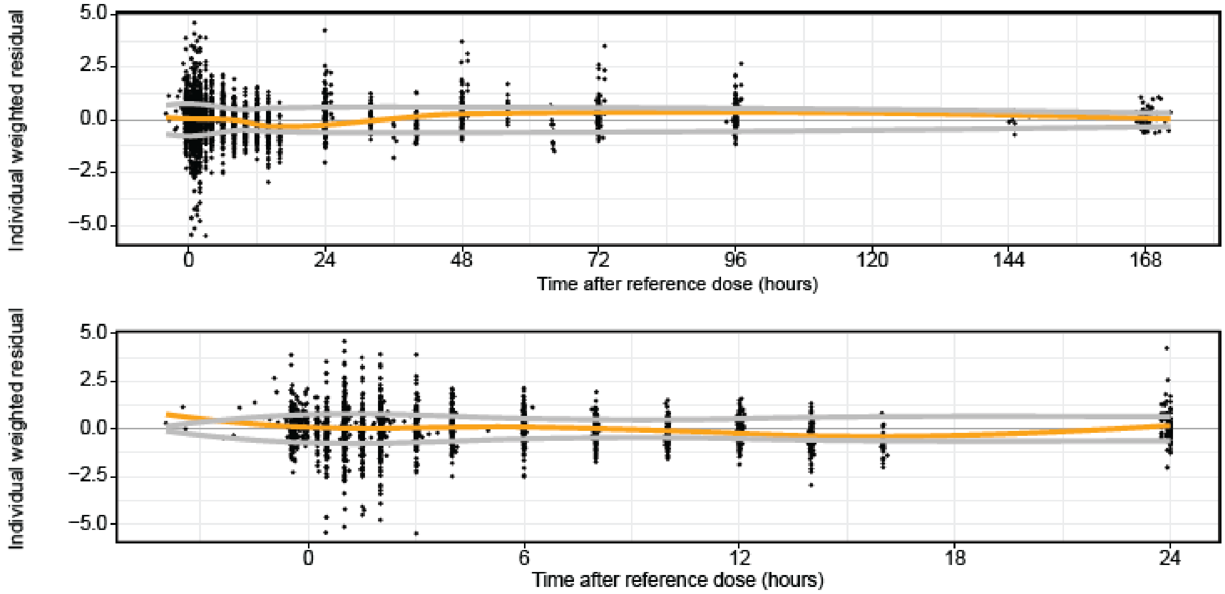

C

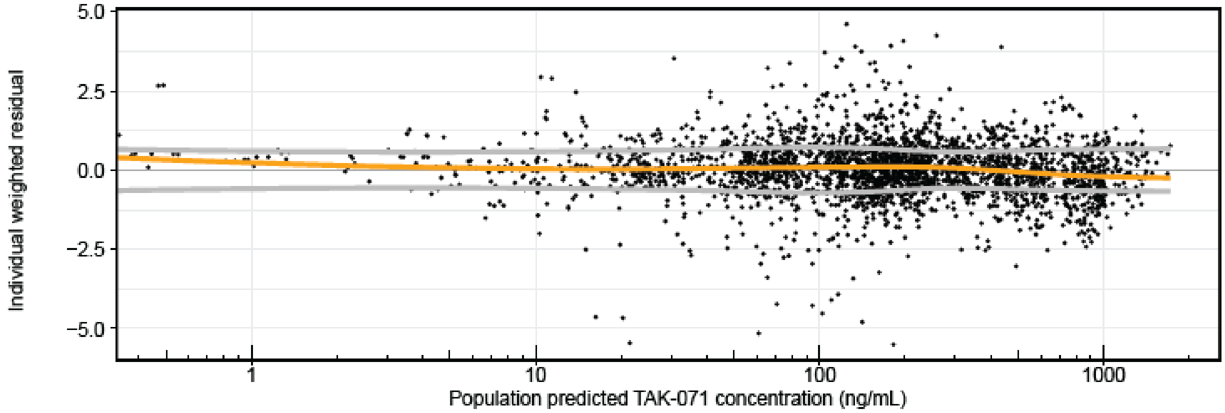

Figure S1. Goodness-of-fit plots for the final population pharmacokinetic model of TAK-071. (A) Scatterplot of observations versus predictions (restricted to observations > lower limit of quantification). Color circles represent the individual data points from the 2 clinical trials. The gray diagonal lines are lines of identity. (B) Plots of individual weighted residuals over time. The black circles represent the data points. The yellow line represents a LOESS regression of the residuals. The gray lines represent the LOESS regression of the absolute and negative absolute residuals. The lower figure in (B) only shows data up to 24 hours post dose. (C) Individual weighted residuals versus population predictions. The black circles represent the individual data points from both clinical trials. The yellow line represents the LOESS regression of the residuals. The gray lines represent the LOESS regression of the absolute and negative absolute residuals. LOESS, locally estimated scatterplot smoothing.

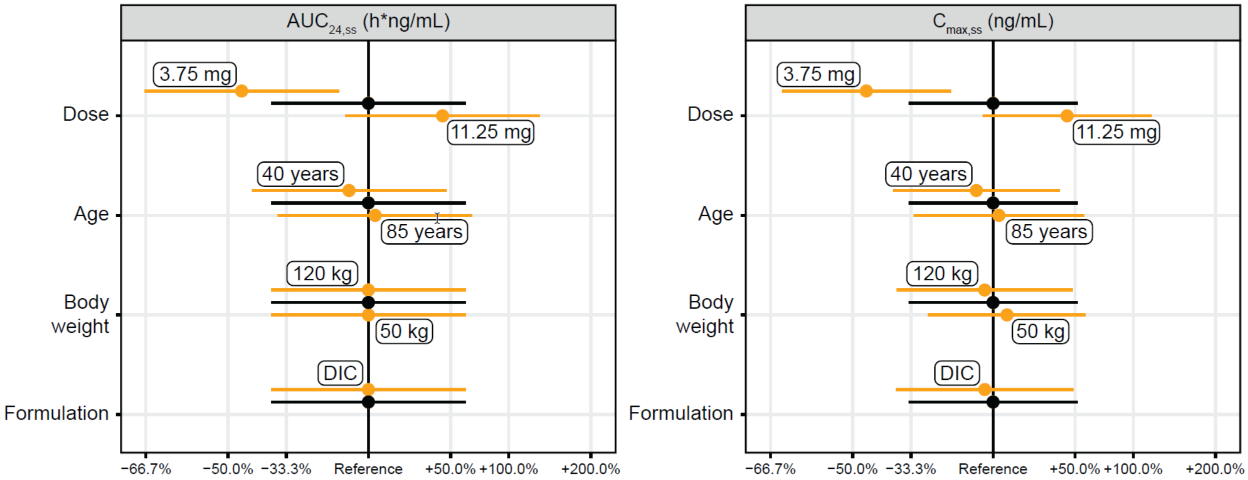

Figure S2. Tornado plots to visualize covariate effects on exposure parameters. Black dots and horizontal lines represent the geometric means and 80% prediction intervals of the pharmacokinetics in the reference population (age 70 years and body weight 80 kg), treated with 7.5-mg TAK-071 once daily in tablet formulation. The yellow dots and horizontal lines represent the geometric means and 80% prediction intervals of the pharmacokinetics in the population with different covariates values (black box), treated with 7.5-mg TAK-071 once daily in tablet formulation. $AUC_{24,ss}$, area under the concentration-time curve over 24 hours at steady sate; $C_{max,ss}$, maximum plasma concentration at steady state; DIC, drug-in-capsule.

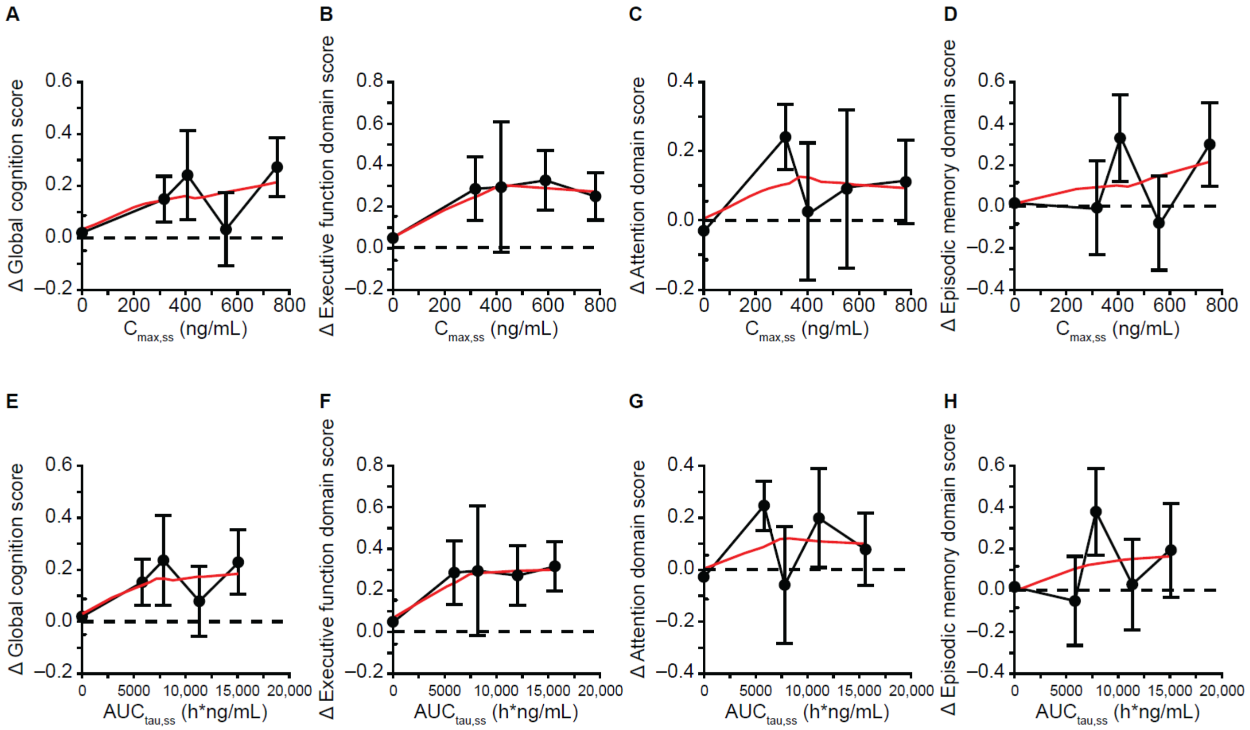

Figure S3. Quartile plots of global cognition score and domain scores versus $C_{max,ss}$ and $AUC_{tau,ss}$. (A) Change from baseline in global cognition score versus $C_{max,ss}$. (B) Change from baseline in executive function domain score versus $C_{max,ss}$. (C) Change from baseline in attention domain score versus $C_{max,ss}$. (D) Change from baseline in episodic memory domain score versus $C_{max,ss}$. (E) Change from baseline in global cognition score versus $AUC_{tau,ss}$. (F) Change from baseline in executive function domain score versus $AUC_{tau,ss}$. (G) Change from baseline in attention domain score versus $AUC_{tau,ss}$. (H) Change from baseline in episodic memory domain score versus $AUC_{tau,ss}$. Mean (± standard error) scores change from baseline following 6 weeks of placebo and each quartile of the $C_{max,ss}$ following 6 weeks of TAK-071 treatment were presented. The red lines represent the locally estimated scatterplot smoothing regression lines of the mean values. Δ, change from baseline; $AUC_{tau,ss}$, area under the concentration-time curve during a dosing interval at steady sate; $C_{max,ss}$, maximum plasma concentration at steady state.

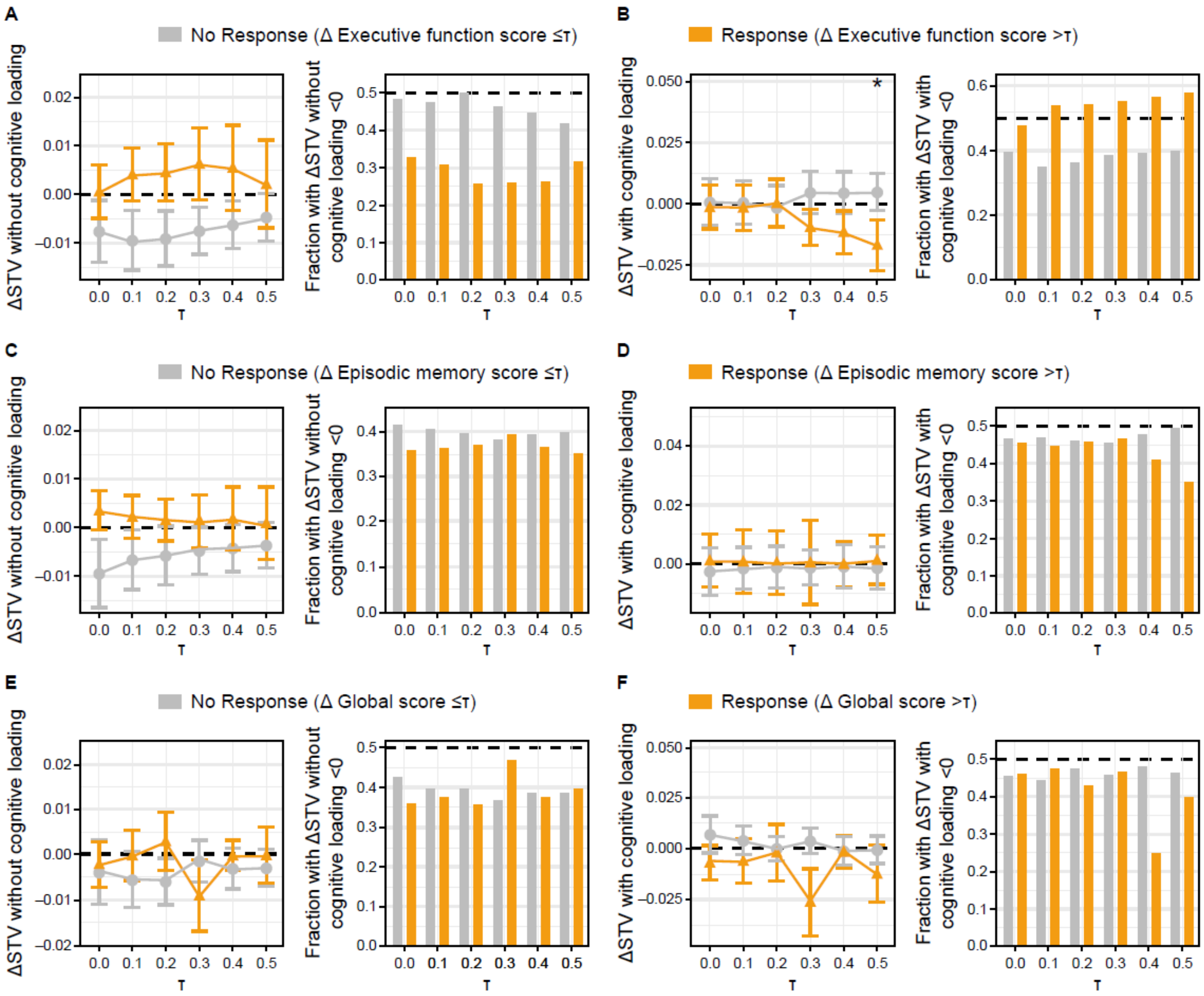

Figure S4. Point estimates and distributions for the ΔSTV for cognitive response and no response groups, which are defined using varying thresholds (τ) applied to (A–B) change from baseline in executive function domain score, (C–D) change from baseline in episodic memory domain score, and (E–F) change from baseline in global score. Values for ΔSTV without cognitive loading are in panels (A), (C), and (E), and those with cognitive loading are in (B), (D), and (F). A response represents an improvement in cognition with respect to the score used, with increasing values of τ indicating greater improvement. The panels on the left display the mean ± standard error, with * indicating statistical significance between the mean ΔSTV of cognitive response group and that for no response of $P < .05$. The panels on the right provide the fraction of data points in the response and no response groups exhibiting an improvement in STV (ie, ΔSTV <0). Δ, change from baseline; STV, stride time variability.

Supplemental Tables

Table S1. Summary of Demographics and Baseline Characteristics of Individuals Included in the Population Pharmacokinetic Analysis

Categorical variables	Level	Number of individuals (%)
Number of individuals		153
Status	Healthy	92 (60.1%)
	Parkinson disease	61 (39.9%)
Sex	Female	18 (11.8%)
	Male	135 (88.2%)
Race	American Indian or Alaska Native	1 (0.7%)
	Asian	19 (12.4%)
	Black or African American	23 (15.0%)
	Multiple	5 (3.3%)
	Native Hawaiian or other Pacific Islander	3 (2.0%)
	Not reported	2 (1.3%)
	White	100 (65.4%)
Japanese or non-Japanese	Japanese	15 (9.8%)
	Non-Japanese	138 (90.2%)
Analysis race	Asian	19 (12.4%)
	Black or African American	23 (15.0%)
	White or Other	111 (72.5%)
**Continuous variable**		**Median (range)**
Age (years)		44 (18–83)
Albumin (g/L)		45 (38–52)
ALT (U/L)		16 (5–66)*
AST (U/L)		19 (7–43)
Bilirubin (μmol/L)		8.55 (3–25.7)**
Body weight (kg)		78.7 (47.3–122)
Creatinine clearance (mL/min/1.73m^2)		106 (56.9–180)
eGFR (mL/min)		92.4 (57.1–143)
Serum creatinine (μmol/L)		83 (53–115)

*One individual's ALT was not available at baseline, which was imputed to 16 U/L.
**Two individuals' bilirubin were not available at baseline, which were imputed to 8.55 μmol/L.

Table S2. Summary of Plasma Pharmacokinetic Parameter Estimates of TAK-071 Following Once-Daily Oral Administration of 5- or 7.5-mg TAK-071 to People With Parkinson Disease: Main Cohort

Day [n]	Parameter	t_{max} (hours)	C_{max} (ng/mL)	C_{trough} (ng/mL)	AUC_{24} (h*ng/mL)
1 [53]	Mean[a]	2.10	132	–	2490
	SD[b]	1.30–12.00	44.8	–	824
	CV%	–	33.9	–	33.1
**Day [n]**		**$t_{max,ss}$ (hours)**	**$C_{max,ss}$ (ng/mL)**	**$C_{trough,ss}$ (ng/mL)**	**$AUC_{tau,ss}$ (h*ng/mL)**
42 [44]	Mean[a]	1.75	507	368	10,000
	SD[b]	1.10-6.00	175	149	3710
	CV%		34.5	40.5	37.1

CV%, percent coefficient of variation; AUC_{24}, area under the concentration-time curve from time 0 to 24 hours; $AUC_{tau,ss}$, area under the concentration-time curve during a dosing interval at steady state; C_{max}, maximum observed plasma concentration; $C_{max,ss}$, maximum observed plasma concentration at steady state; C_{trough}, plasma concentration at the end of a dosing interval; $C_{trough,ss}$, plasma concentration at the end of the dosing interval at steady state; SD, standard deviation; t_{max}, time to reach C_{max}; $t_{max,ss}$, time to reach $C_{max,ss}$.

[a]Median is presented for t_{max} and $t_{max,ss}$.

[b]Minimum–maximum is presented for t_{max} and $t_{max,ss}$.

Table S3. Summary Table Providing the Number of Data Points in the Response and No Response Groups Defined Using Thresholds (τ) Applied to the Different Change From Baseline Cognition Scores and Effect Size With Respect to STV

Cognition score	Noncognition endpoint	τ	Participants, n		Effect size[a]	Apparent *P* value[b]	Statistical significance
			Response	No response			
Δ Global	ΔSTV without cognitive loading	0	48	28	0.4792	.62	ns
		0.1	39	37	0.4699	.68	ns
		0.2	27	49	0.4550	.74	ns
		0.3	14	62	0.5749	.19	ns
		0.4	7	69	0.5487	.34	ns
		0.5	4	72	0.5486	.38	ns
	ΔSTV with cognitive loading	0	43	31	0.5964	.081	ns
		0.1	34	40	0.5853	.106	ns
		0.2	24	50	0.5283	.349	ns
		0.3	12	62	0.6263	.085	ns
		0.4	6	68	0.4730	.59	ns
		0.5	3	71	0.6338	.221	ns
Δ Attention	ΔSTV without cognitive loading	0	34	30	0.6235	.0457	*
		0.1	23	41	0.6554	.0202	*
		0.2	16	48	0.7357	.0022	**
		0.3	12	52	0.6859	.0235	*
		0.4	8	56	0.7366	.0161	*
		0.5	8	56	0.7366	.0161	*
	ΔSTV with cognitive loading	0	31	31	0.4839	.59	ns
		0.1	19	43	0.4688	.65	ns
		0.2	13	49	0.4741	.61	ns
		0.3	9	53	0.4507	.68	ns
		0.4	6	56	0.6042	.21	ns
		0.5	6	56	0.6042	.21	ns

Δ Executive function	ΔSTV without cognitive loading	0	41	32	0.4169	.89	ns
		0.1	35	38	0.3820	.96	ns
		0.2	32	41	0.3605	.98	ns
		0.3	24	49	0.3741	.96	ns
		0.4	20	53	0.4038	.9	ns
		0.5	18	55	0.4313	.81	ns
	ΔSTV with cognitive loading	0	40	30	0.5400	.288	ns
		0.1	37	33	0.5405	.283	ns
		0.2	33	37	0.5536	.223	ns
		0.3	25	45	0.5796	.139	ns
		0.4	21	49	0.5918	.115	ns
		0.5	17	53	0.6360	.047	*
Δ Episodic memory	ΔSTV without cognitive loading	0	41	35	0.4230	.88	ns
		0.1	35	41	0.4390	.82	ns
		0.2	34	42	0.4587	.73	ns
		0.3	27	49	0.4754	.64	ns
		0.4	21	55	0.4710	.65	ns
		0.5	19	57	0.4894	.56	ns
	ΔSTV with cognitive loading	0	36	38	0.5409	.28	ns
		0.1	30	44	0.5515	.23	ns
		0.2	29	45	0.5739	.14	ns
		0.3	23	51	0.5772	.15	ns
		0.4	18	56	0.5308	.35	ns
		0.5	16	58	0.5097	.46	ns

Δ, change from baseline; ns, not significant; STV, stride time variability.

[a]In this setting, the effect size, which is measured using Vargha and Delaney's A, represents the probability that an observed ΔSTV (without or with cognitive loading) for the cognitive response group is less than the observed ΔSTV (without or with cognitive loading) for the no response group.

[b]Apparent *P* values were computed using the Wilcoxon rank-sum test for the scenario that the mean ΔSTV (without or with cognitive loading) for response group is less than that for no response.

$^{*}P$ value <0.05; $^{**}P$ value <0.01.

Supplemental Equations

Supplemental Equation 1. Equations for exposure–response models

Linear shape model: $E = slope * C_{trough,ss}$

Emax shape model: $E = E_{max} * C_{trough,ss}/(C_{trough,ss} + EC_{50})$

Reverse "U" shape model: $E = E_{max} * (C_{trough,ss}^{Hill} / (C_{trough,ss}^{Hill} + EC_{50}^{Hill}) – C_{trough,ss}^{Hill} / (C_{trough,ss}^{Hill} + IC_{50}^{Hill}))$

Where $C_{trough,ss}$ = plasma concentration at the end of the dosing interval at steady state; EC_{50} = half maximal effective concentration; E = response; E_{max} = maximal effect; IC_{50} = half maximal inhibition concentration; I_{max} = maximal inhibition; Hill = Hill coefficient related to the sigmoidal shape.

Supplemental Equation 2. Equation of interindividual variability (quantified using CV%) for a log-normal distributed random-effect parameter

$$CV = \sqrt{e^{\omega^2} - 1}$$

where ω^2 is the variance of the respective between subject variability (element of the OMEGA matrix). CV = coefficient of variation.

Supplemental Code. NONMEM Control Stream of the Final Model

```
$PROBLEM executed with MeRmd2
$INPUT STUDY STATUS ID TRT PERIOD ADOSE DOSE VISIT DAY NTAD TIME TAD EVID
AMT
      FORM DV MDV BLQ LLOQ AGE SEX RACE WEIGHT ALB ALT AST BILI CREAT
JAPANESE
      CLCR EGFR SAMPLING EXCL ARACE EXCL2
$DATA data.csv IGNORE=@
$SUBROUTINE ADVAN5

$MODEL
  COMP = (GUT     DEFDOSE)
  COMP = (TR1     )
  COMP = (TR2     )
  COMP = (ABS1    )
  COMP = (ABS2    )
  COMP = (CENTRAL DEFOBS)

$PK
  ; FREL {label="Rel. bioavailability (%)"}
  TVFREL = THETA(1)
  TVFREL = TVFREL * (DOSE/5)**THETA(14)
  FREL   = TVFREL

  F1 =  FREL/100

  NT   =  3

  ; MTT {label="Mean transit time (h)"}
  ; we model the meant transit time as sum of Tlag + MTT
  ALAG1 = THETA(2)
  TVMTT = THETA(3) - ALAG1
  MTT   = TVMTT * EXP(ETA(1))

  KTR = (NT+1)/MTT

  ; FRAC  {label="Fraction absorbed slowly (%)"}
  ; FRAC_ {label="Fraction absorbed slowly (logit)"}
  TVFRAC  = THETA(4)/100
  TVFRAC_ = LOG(TVFRAC/(1-TVFRAC))
  IF( FORM == 2 ) TVFRAC_ = TVFRAC_ * THETA(15)
  FRAC_   = TVFRAC_ + ETA(2)
  IF( SAMPLING == 2 ) FRAC_ = TVFRAC_
  FRAC    = 100 * EXP(FRAC_) / (1 + EXP(FRAC_))

  ; KA {label="Absorption rate (1/h)"}
  TVKA = THETA(5)
  IF( FORM == 2 ) TVKA = TVKA * THETA(13)
  TVKA = TVKA * (DOSE/5)**THETA(12)
  KA   = TVKA * EXP(ETA(3))
```

```
  IF( SAMPLING == 2 ) KA = TVKA

  ; KAQ {label="Absorption ratio quick/slow"}
  TVKAQ = THETA(6)
  KAQ   = TVKAQ * EXP(ETA(4))

  ; CL {label="Elim. clearance (L/h)"}
  TVCL = THETA(7)
  TVCL = TVCL * (AGE/70)**THETA(17)
  CL   = TVCL * EXP(ETA(5))

  ; VC {label="Centr. volume (L)"}
  TVVC = THETA(8)
  TVVC = TVVC * (WEIGHT/80)**THETA(16)
  VC   = TVVC * EXP(ETA(6))

  S6 = VC/1000

  K1T2 = KTR
  K2T3 = KTR
  K3T4 = KTR * FRAC/100
  K3T5 = KTR * (1 - FRAC/100)
  K4T6 = KA
  K5T6 = KA * KAQ
  K6T0 = CL/VC

$ERROR
  IPRED = F

  W = 1
  IF( IPRED > 0 ) W = SQRT((THETA(9)/100*IPRED)**2 + THETA(10)**2)
  IF( SAMPLING == 2 ) W = W * THETA(11)

  IRES  = DV - IPRED
  IWRES = IRES/W

  ; M3-Method
  Y = 0
  IF( BLQ == 0) THEN
    F_FLAG = 0
    Y = IPRED + W*EPS(1)
  ELSE
    F_FLAG = 1
    Y = PHI((LLOQ-IPRED)/W)
  ENDIF


$THETA
 100          FIX ; FREL          {param="Rel. bioavailability",     role="TV",          unit="%"}
 0.2          FIX ; TLAG          {param="Lag time",                 role="TV",          unit="h"}
 (0.2, 1.52)      ; MTT           {param="Mean transit time",        role="TV",          unit="h"}
```

```
 (0, 79.97, 100)     ; FRAC             {param="Fraction absorbed slowly",    role="TV",
unit="%"}
 (0, 0.8875)         ; KA               {param="Absorption rate",             role="TV",
unit="1/h"}
 (2, 70.29)          ; KA.Q             {param="Absorption ratio quick/slow", role="TV",
unit="ratio"}
 (0, 0.5866)         ; CL               {param="Clearance",                   role="TV",               unit="L/h"}
 (0, 45.81)          ; VC               {param="Volume",                      role="TV",               unit="L"}
 (0, 11.6)           ; WP               {param="RUV",                         role="prop.",            unit="%"}
 (0, 5.272)          ; WA               {param="RUV",                         role="add.",
unit="ng/mL"}
 (0, 1.169)          ; WS               {param="RUV",                         role="sparse",           unit="ratio"}
 -0.8346             ; KA~DOSE          {param="Absorption rate",             role="Dose-effect",
unit="power"}
 (0, 25.89)          ; KA~FORM=Tablet   {param="Absorption rate",             role="Tablet-effect",
unit="ratio"}
 -0.1082             ; FREL~DOSE        {param="Rel. bioavailability",        role="Dose-effect",
unit="power"}
 (0, 0.4065)         ; FRAC~FORM=Tablet {param="Fraction absorbed slowly",    role="Tablet-
effect",      unit="ratio"}
 0.8228              ; VC~WEIGHT        {param="Volume",                      role="Body weight-effect",
unit="power"}
 -0.3104             ; CL~AGE           {param="Clearance",                   role="Age-effect",
unit="power"}

$OMEGA
 0.3771      ; MTT  {param="Mean transit time",           role="BSV", dist="LN"}
 1.269       ; FRAC {param="Fraction absorbed slowly",    role="BSV", dist="logit"}
 0.5869      ; KA   {param="Absorption rate",             role="BSV", dist="LN"}
 0       FIX ; KA.Q {param="Absorption ratio quick/slow", role="BSV", dist="LN"}
 0.1303      ; CL   {param="Clearance",                   role="BSV", dist="LN"}
 0.05363     ; VC   {param="Volume",                      role="BSV", dist="LN"}

$SIGMA
 1 FIX ; RV {param="Residual variability", role="WSV", dist="N"}

$ESTIMATION      METHOD=COND INTERACTION MAXEVAL=9999 POSTHOC NOABORT
SIGDIGITS=3 PRINT=1 SORT LAPLACE ETASTYPE=1
$COVARIANCE      MATRIX=S UNCONDITIONAL
$TABLE
  STUDY STATUS ID TRT PERIOD ADOSE DOSE VISIT DAY NTAD TIME TAD EVID AMT
FORM DV
  MDV BLQ LLOQ AGE SEX RACE WEIGHT ALB ALT AST BILI CREAT JAPANESE CLCR
EGFR
  SAMPLING EXCL ARACE EXCL2 TVFREL FREL F1 NT ALAG1 TVMTT MTT KTR TVFRAC
FRAC
  TVKA KA TVKAQ KAQ TVCL CL TVVC VC S6 K1T2 K2T3 K3T4 K3T5 K4T6 K5T6 K6T0 W
IRES
  IWRES ETA1 ETA2 ETA3 ETA4 ETA5 ETA6 IPRED PRED WRES CWRES CIWRES NPDE
  FILE=autotable.txt
  NOTITLE ONEHEADER NOPRINT NOAPPEND UNCONDITIONAL
```
